# Silver Decoration of Vertically Aligned MoS_2_-MoO_x_ Nanosheets: A Comprehensive XPS Investigation

**DOI:** 10.3390/ma17122882

**Published:** 2024-06-13

**Authors:** Khaled Al Youssef, Arkaprava Das, Jean-François Colomer, Axel Hemberg, Xavier Noirfalise, Carla Bittencourt

**Affiliations:** 1 Chimie des Interactions Plasma-Surface (ChIPS), Materials Institute, University of Mons, 23 Place du Parc, 7000 Mons, Belgium; khaled.alyoussef@umons.ac.be; 2 Institute of Physical and Theoretical Chemistry, University of Tuebingen, 72076 Tuebingen, Germany; arkapravadas222@gmail.com; 3 Research Group on Carbon Nanostructures (CARBONNAGe), University of Namur, 61 Rue de Bruxelles, 5000 Namur, Belgium; jean-francois.colomer@unamur.be; 4 Materia Nova, 3 Avenue Copernic, 7000 Mons, Belgium; axel.hemberg@materianova.be (A.H.); xavier.noirfalise@materianova.be (X.N.)

**Keywords:** MoS_2_, vertically aligned, p-type, silver nanoparticles, functionalization, plasma deposition, nucleation sites, XPS, valence band

## Abstract

This study investigates the simultaneous decoration of vertically aligned molybdenum disulfide nanostructure (VA-MoS_2_) with Ag nanoparticles (NPs) and nitrogen functionalization. Nitrogen functionalization was achieved through physical vapor deposition (PVD) DC-magnetron sputtering using nitrogen as a reactive gas, aiming to induce p-type behavior in MoS_2_. The utilization of reactive sputtering resulted in the growth of three-dimensional silver structures on the surface of MoS_2_, promoting the formation of silver nanoparticles. A comprehensive characterization was conducted to assess surface modifications and analyze chemical and structural changes. X-ray photoelectron spectroscopy (XPS) showed the presence of silver on the MoS_2_ surface. Scanning electron microscopy (SEM) confirmed successful decoration with silver nanoparticles, showing that deposition time affects the size and distribution of the silver on the MoS_2_ surface.

## 1. Introduction

Over the past two decades, Molybdenum disulfide (MoS_2_), a standout compound among transition metal dichalcogenides (TMDs), has attracted significant interest. Its crystal structure exhibits multiple phases, including the semiconducting 2H-MoS_2_ phase, which contains inherent sulfur vacancies within its lattice, classifying it as an n-type semiconductor [1,2,3]. MoS_2_ has been the subject of extensive study due to its intriguing optical and electronic properties. The morphology of MoS_2_ has emerged as a critical factor influencing its performance in various practical applications. Recently, a few layers of vertically aligned MoS_2_ (VA-MoS_2_) have demonstrated exceptional performance in diverse applications due to their predominantly exposed edges with high surface energy [4,5,6]. However, these edges are inherently unstable, making VA-MoS_2_ suitable for applications involving functionalization [7,8]. The vertically aligned morphology of MoS_2_ is believed to provide more active sites when decorated with Ag nanoparticles (NPs) compared to horizontally aligned or randomly arranged MoS_2_ nanosheets. This increase is attributed to the three-dimensional (3D) architecture of the VA-MoS_2_ structure, which is rich in under-coordinated atomic sites. The 3D morphology offers a large effective area that can accommodate a higher density of metal-decorated active sites for analyte interaction [9]. The interplay between Ag(NPs) and MoS_2_ confers enhanced chemical stability for different applications, including in Surface-enhanced Raman Spectroscopy (SERS) detection [8]. The concurrent incorporation of nitrogen atoms and silver addition into molybdenum disulfide (MoS_2_) nanostructures is anticipated to synergistically passivate the exposed edges, modulate the electronic structure, and stabilize the overall nanostructured system [10].

Given the unique characteristics and potential applications of MoS_2_, a variety of methods have been employed for its synthesis. Among these methods, chemical vapor deposition (CVD) has emerged as an important technique for the synthesis of high-quality vertically aligned MoS_2_ (VA-MoS_2_). The CVD synthesis process comprises two main steps: the initial deposition of a thin Mo film followed by sulfurization [11]. Recent research indicates that the thickness of the Mo thin film plays a pivotal role in the growth mechanism, determining whether the resulting substrate exhibits a planar or vertical orientation. Notably, studies have revealed a critical threshold at a thickness of 5 nm, where films exceeding this value tend to grow predominantly vertically, while Mo films thinner than 5 nm lead to planar-oriented substrates [12].

The decoration of MoS_2_ with metal nanoparticles (NPs) aims to enhance the performance and functionality of MoS_2_ in various applications, particularly in catalysis and sensing technologies [13]. The interaction between metal nanoparticles and MoS_2_ increases chemical stability and provides additional active sites for improved reactivity and sensitivity [14]. Noble metals such as silver nanoparticles (Ag(NPs)) are distinguished for their unique plasmonic antenna characteristics, significantly amplifying the optical response of the MoS_2_ towards different molecules (analytes) in SERS detection [15,16]. Free electrons in the conduction band of Ag particles trigger surface plasmon resonance (SPR), resulting in enhanced scattered signal through localized surface plasmon resonance (LSPR) upon interaction with incident light. The decoration of MoS_2_ with Ag(NPs) increases the surface-to-volume ratio and the surface energy, thereby facilitating enhanced interactions between the MoS_2_ and different molecules [5].

Among the methodologies used for the deposition of metal nanoparticles on a substrate, direct-current (DC)-magnetron sputtering stands out as a prominent technique. This method, which employs cold plasma, is widely recognized for its environmentally friendly nature and cost-effectiveness, with minimal generation of byproducts [17]. Notably, this technique enables the deposition of metal nanoparticles on nanostructured films without altering the original morphology [18]. For instance, Ag(NPs) can be deposited onto a nanostructured film directly from an Ag target, ensuring no oxidation or intermediate reactions occur during the deposition process. The presence of Ag(NPs) has been observed to induce a shift in the Fermi level of MoS_2_ towards the valence band (VB) while simultaneously lowering the conduction band minimum (CBM), introducing impurity levels between the valence band maximum (VBM) and CBM, resulting in a p-type MoS_2_-Ag-doped material [10,19].

This study focuses on the simultaneous decoration of VA-MoS_2_ with Ag(NPs) and functionalization with nitrogen chemical species. The functionalization was performed using physical vapor deposition (PVD) DC-magnetron sputtering with nitrogen as a reactive gas. The functionalization with nitrogen is expected to lead to p-type MoS_2_ [20,21]. Additionally, using reactive sputtering leads to the growth of three-dimensional silver structures on the MoS_2_ nanostructure surface, facilitating the formation of silver nanoparticles [20,21]. The decoration with silver was performed for three different silver deposition times: 5, 10, and 15 s. For comparison, a commercial powder of MoS_2_ underwent the same functionalization with varying functionalization times of 5, 10, 15, 20, and 40 min. A comprehensive set of characterizations was performed to examine surface modifications and evaluate chemical and structural alterations. These characterizations included scanning electron microscopy (SEM), Raman Spectroscopy, and X-ray photoelectron spectroscopy (XPS).

## 2. Materials and Methods

### 2.1. Chemicals

A commercially available bulk MoS_2_ powder with a particle size of less than 2 μm from Sigma-Aldrich (99% purity, molecular weight 160.07 g/mol) was used as the standard. The Ag target used for the deposition of silver on the MoS_2_ surface was purchased from advanced engineering materials with a diameter of 50.8 mm and a purity of 99.99%. The nitrogen used as reactive gas was from Air Liquide, and the purity was N_2_ ≥ 99.999%.

### 2.2. Synthesis of Vertically Aligned MoS_2_

The growth of MoS_2_ nanosheets was achieved through a sulfurization process using an atmospheric pressure chemical vapor deposition (CVD) technique, as reported elsewhere [22]. A 50 nm thick Mo film was deposited onto a SiO_2_/Si substrate using DC-magnetron sputtering (Quorum, Q15T/ES, Laughton, East Sussex, UK). Argon (99.9995% purity) was used to sputter a Mo target. The Argon pressure within the deposition chamber was 1 × 10^−3^ mbar. A quartz tube reactor was employed for the sulfurization process. The SiO_2_/Si substrate with the Mo film was placed into the reactor along with S powder, which was distributed into two predefined zones of the tube (zone 1 at 40 °C and zone 2 at 850 °C) to ensure proper temperature distribution upon insertion of the reactor into the furnace. After completion of the reaction, the quartz tube was removed from the reactor and cooled for 1 h under an Argon flow.

### 2.3. Silver Deposition and Functionalization of the Samples

An RPG-50 pulsed DC plasma generator from ENI ( Roma, Italy) was used for sputtering. The background pressure was 10^−7^ Torr. The working voltage ranged from 390 to 420 V, with a current of approximately 0.1 A. The sputtering yield of Ag was 3.12 atoms per ion (Ag atom per Ar ion). To achieve uniform deposition of Ag(NPs) onto the powder particles, the commercial MoS_2_ powder underwent slight vibration [23].

The samples, commercial powder MoS_2_ and VA-MoS_2_, were individually introduced into the chamber equipped with the pulsed DC-magnetron sputtering chamber. Both sets of samples were subjected to the same treatments. The chamber base pressure was ~4 × 10^−6^ mbar. For the Ag sputtering, a mixture of Argon (Ar) and nitrogen (N_2_) as the reactive gas and a silver target were used. The functionalization parameters used in all experiments were as follows: average power (P) = 40 W, gas flux (Φ) = 20 sccm (Φ(Ar) = 2 sccm and Φ(N_2_) = 18 sccm), the working pressure (P_w_) = 30 mTorr, and functionalization time (t) = 5–10–15 s for VA-MoS_2_ and 5–10–15–20–40 min for the commercial powder MoS_2_.

Functionalized samples are labeled as follows: for the powder samples, N-MoS_2_ (Ag_x_), and for the vertically aligned samples, N-VA-MoS_2_ (Ag_x_), where N represents nitrogen, x is the deposition time used to dope the samples, and VA is an abbreviation of vertically aligned.

### 2.4. Characterization of the Samples

X-ray photoelectron spectroscopy (XPS) was used to identify the chemical elements and their chemical environment in the sample. X-ray photoelectron spectroscopy (XPS) analysis of the samples was carried out in the VERSAPROBE PHI 5000 instrument from Physical Electronics USA equipped with an aluminum anode that emitted Kα X-ray radiation with an energy of 1486.6 eV. The energy resolution was 0.6 eV. A dual-beam charge neutralization (electron gun (~1 eV) and Argon ion gun (<10 eV)) was used for charge compensation. The measurements were performed at a takeoff angle of 45° using a hemispherical electron energy analyzer. CasaXPS software (version 2.3.25) was used for data analysis.

Scanning electron microscopy (SEM) imaging was performed using two instruments: the JEOL-JSM-7500F field-emission scanning electron microscope operated at 2 kV and the FESEM-SU8020-HITACHI with triple detectors, including a Thermo Scientific NORAN System 7 X-ray detector operated at 3 kV and 5 kV.

The nanoparticle (NP) size distribution was assessed using ImageJ software (version 1.51) [24] and applied to SEM images. Approximately 200 NPs were analyzed in randomly selected regions, and the individual NP diameters were measured. The Gaussian fitting of the size distribution data provided the average NP size for each sample.

## 3. Results and Discussion

### 3.1. Scanning Electron Microscopy (SEM)

**Figure 1:**
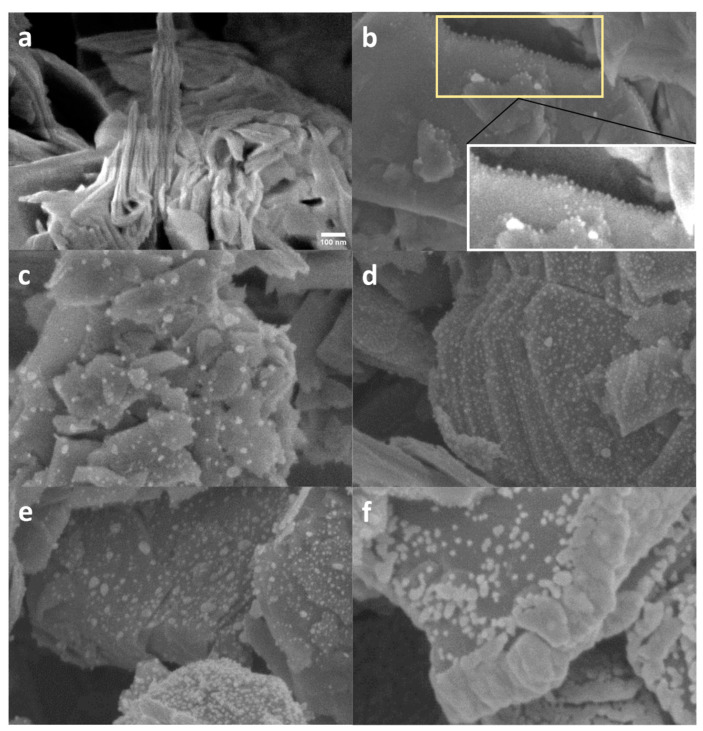
SEM images of the pristine and the functionalized MoS_2_ powder samples for (**a**) pristine, (**b**) 5 min, yellow frame shows an edge decorated with particles, white frame is a magnification of the yellow frame, (**c**) 10 min, (**d**) 15 min, (**e**) 20 min, and (**f**) 40 min. The scale bar in Figure 1a is applicable across all the panels of the figure.

The morphology of the MoS_2_ commercial powder sample is characterized by MoS_2_ platelets (Figure 1a). After functionalization, distinct bright spots become apparent on the surface of the platelets, indicating the presence of dispersed Ag(NPs) (Figure 1). These Ag(NPs) are predominantly situated at the edges of the platelets, as highlighted in Figure 1b (inset). Increasing silver deposition time, the number of NPs and their size increase. Upon 40 min of functionalization, the Ag(NPs) tend to coalesce, preferentially localized at the edge of the platelets (Figure 1f).

**Figure 2:**
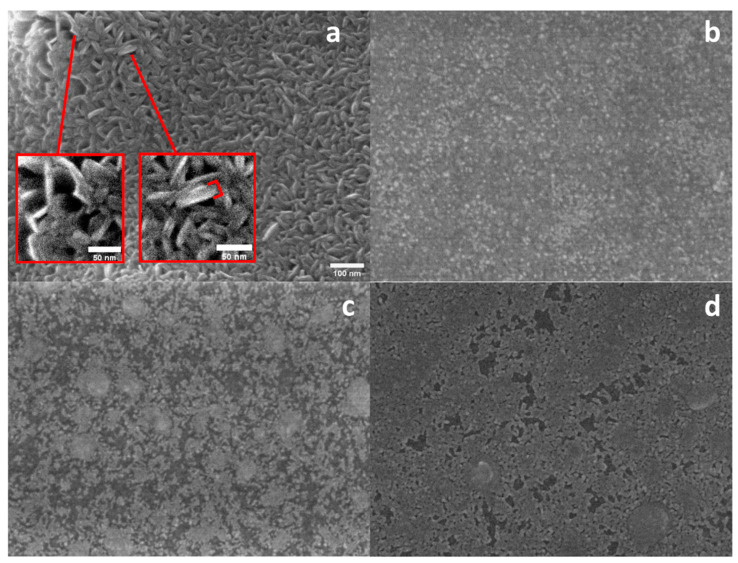
SEM image of (**a**) the as-synthesized VA-MoS_2_, and the functionalized VA-MoS_2_ samples decorated with Ag(NPs) for (**b**) 5 s, (**c**) 10 s, (**d**) 15 s. The scale bar in Figure 2a is applicable across all the panels of the figure. Red frames in (**a**) magnification showing nanoplatelets.

The scanning electron microscopy (SEM) image of the as-synthesized VA-MoS_2_ shows a uniform distribution of nanoplatelets that can be described as vertically aligned nanosheets (Figure 2). The vertical alignment is illustrated in Figure S1, displaying an image of a sample cross-section where aligned nanoplatelets can be observed.

The magnified view in the inset of Figure 2a reveals these platelets with an average thickness of about 18.7 nm, corresponding to an average of 27 layers of MoS_2_, considering each layer of MoS_2_ to be 0.65 nm thick [25]. The presence of silver on the surfaces of the functionalized samples is evident from the bright spots observed in Figure 2b–d. Notably, the morphology observed for VA-MoS_2_ demonstrates a similar trend to the powder sample with Ag(NPs) shaping on a flake-like structure as the deposition time increases (Figure 2d). The control of the size and shape of the silver nanoparticles is crucial to optimize the MoS_2_–Ag(NPs) performance as active material in different applications [26]. Regarding the shape, spherical NPs with 50 nm of diameter were reported to yield a significant impact when applied to SERS detection [27,28], while the optimal size range for higher performance is between 10 and 100 nm [29,30]. We have calculated the average size of the nanoparticles on the surface of the different samples (Table 1).

**Figure 3:**
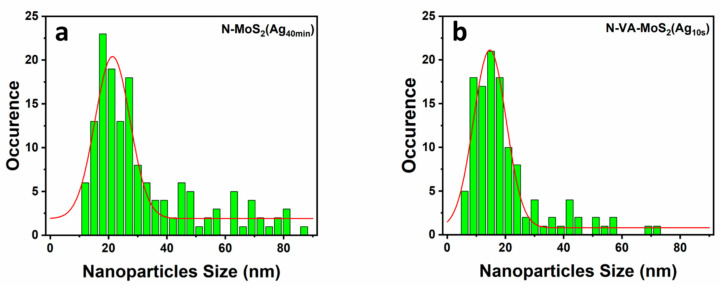
The nanoparticle size distribution was taken from SEM data, with the arithmetic mean shown on each graph and their Gaussian fitting plotted in red. (**a**) N-MoS_2_(Ag_40min_) and (**b**) N-VA-MoS_2_(Ag_10s_).

A silver nanoparticulated thin film was formed on the VA sample functionalized for 15 s. Figure 3 shows the size distribution of silver particles on samples N-MoS_2_ (Ag40min) and N-VA-MoS_2_ (Ag15s). The dispersion can be fitted with a Gaussian. It is worth noting that the average nanoparticle size increases with the deposition time (Table 1).

The process of silver particle formation on the MoS_2_ surface comprises multiple sequential stages, encompassing adsorption, diffusion, agglomeration, nucleation, and growth. When subjected to DC sputtering, silver atoms are physically ejected from a silver target due to bombardment by energetic Ar ions, being deposited onto the surface of MoS_2_. Upon deposition on the MoS_2_ surface, the silver atoms undergo adsorption. The adsorption process of silver atoms on the surface of MoS_2_ is influenced by surface morphology, topographic defects, and the kinetic energy of the deposited atoms [31]. The interaction between silver atoms and the pristine MoS_2_ surface is primarily governed by dispersion forces, as reported in the literature [32]. Following adsorption, the silver atoms may diffuse across the MoS_2_ surface, driven by thermal energy. During diffusion, these silver atoms tend to agglomerate with neighboring atoms, forming small clusters or nuclei. The presence of topographic defects on the MoS_2_ surface can act as active nucleation sites for silver atoms due to their enhanced activity compared to an ideal surface [33], thereby promoting the formation of nuclei. This phenomenon can lead to the localized growth of silver particles near defective regions, such as along the edges of flakes. The presence of defects can impact the agglomeration behavior of silver atoms, resulting in the formation of either larger or smaller clusters based on the specific interaction between the defect and the silver atoms [34]. A longer silver deposition time results in a higher load of NPs with larger sizes, thereby decreasing the space between particles, as we can see for N-VA-MoS_2_ (Ag_15s_) (Figure 2d). Tuning particle size is important in several applications, including SERS signal enhancement [35].

### 3.2. X-ray Photoelectron Spectroscopy

**Figure 4:**
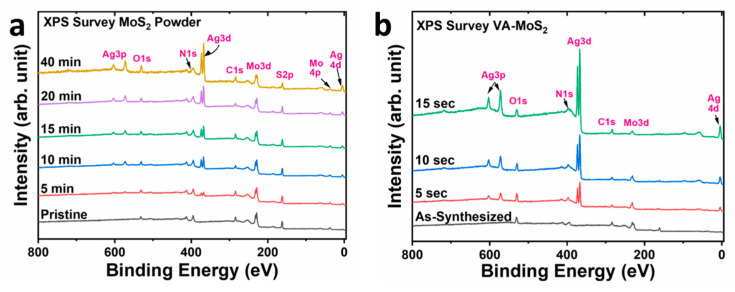
XPS survey spectra of (**a**) powder and (**b**) vertically aligned samples.

A detailed chemical surface characterization of the pristine commercial MoS_2_ powder, the as-synthesized VA-MoS_2_, and their functionalized counterparts was performed using X-ray photoelectron spectroscopy. The XPS survey spectra of these samples are shown in Figure 4.

All binding energies refer to the C1s peak centered at 284.8 eV [36] and normalized with respect to the Mo3d peak. Characteristic core-level peaks Mo3d_5/2_, S2p_3/2_, and Mo3p_3/2_ are centered at ~229.0 eV, ~162.0 eV, and ~395.0 eV, respectively [37]. In the case of the as-synthesized VA-MoS_2_, an additional peak centered at 401 eV, overlapping with the Mo3p_3/2_ peak, indicates the presence of nitrogen introduced during sample synthesis. The intensity of the N1s peak increased in both powder and vertically aligned samples with prolonged functionalization time. The successful surface modification of the MoS_2_ samples with Ag(NPs) is evidenced by the emerging peaks centered at 368 eV, corresponding to Ag3d_5/2_ [38]. Upon comparison of the XPS spectra obtained from the MoS_2_ powder and VA-MoS_2_ samples after functionalization, it is evident that the quantity of Ag(NPs) present in the vertically aligned samples is relatively higher. This is evidenced by the increased relative intensity observed in the Ag peaks, despite the shorter deposition period employed for doping the VA samples. This difference is attributed to the sample morphology. VA-MoS_2_ samples exhibit more exposed edges, offering more active sites for silver nucleation than the powder samples. In addition, during the decoration and functionalization, the powder vibration exposes different regions of the powder, and therefore different nanostructures, to the plasma.

**Figure 5:**
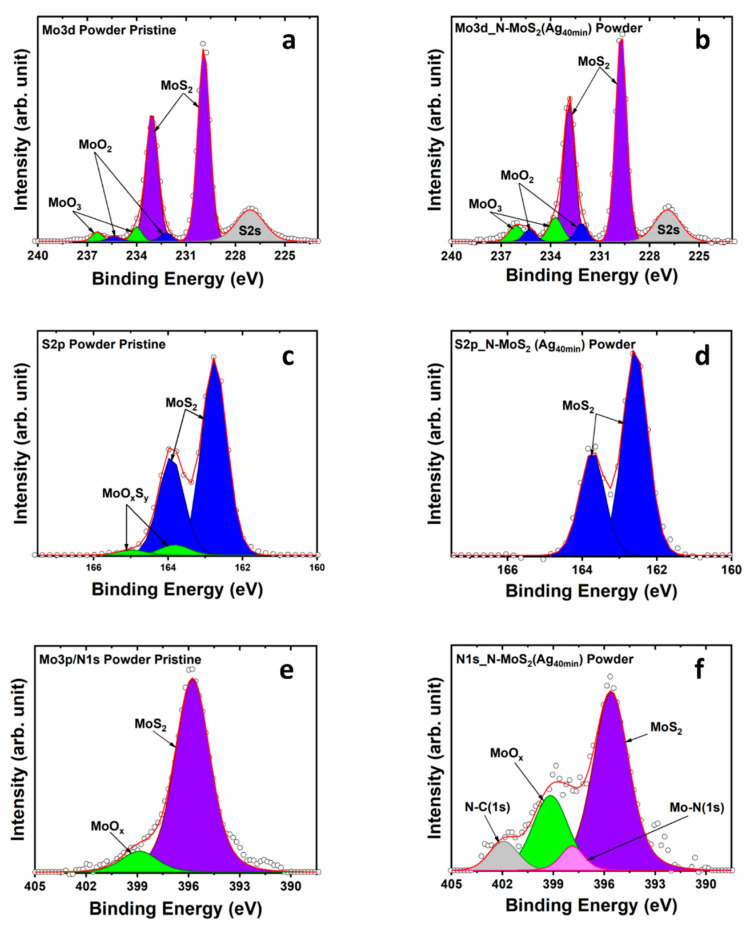
XPS core level of Mo3d, S2p, and N1s regions of MoS_2_ powder, the pristine and the functionalized N-MoS_2_ (Ag_40min_). (**a**,**b**) Mo3d region, (**c**,**d**) S2p region, and (**e**,**f**) N1s region.

X-ray photoelectron spectroscopy, core-level analysis, was carried out to examine the chemical environment of the different elements on the surface of the samples. Three replicate measurements were conducted on each sample to evaluate the reproducibility of the XPS Data. The data exhibited consistent reproducibility across all samples. The precision of each measurement was assessed and shown in Table 2. The deconvolution procedure used Gaussian–Lorentzian (GL) functions to analyze the spectral data. The Full Width at Half Maximum (FWHM) of the GL functions was restricted to a maximum of 2 eV. Additionally, the separation between the two components was set at 3.15 eV for Mo 3d and 6.0 eV for Ag 3d, while the area of the components 3d 3/2 and 3d 5/2 was constrained to 0.6 eV. The background used was Shirley. For the commercial pristine MoS_2_ powder (Figure 5), the Mo3d region was deconvoluted into one singlet and three doublets. The singlet component is correlated with the S2s peak centered at ~227 eV. The semi-conducting phase 2H-MoS_2_ is characterized by the doublet with components centered at 229.9 for Mo3d_5/2_ and 233.1 eV for Mo 3d_3/2_ [39]. Furthermore, the doublet with components centered at 232.2 and 235.3 eV for Mo3d_5/2_ and Mo 3d_3/2_, respectively [40], is correlated with the presence of molybdenum dioxide (MoO_2_). Additionally, the tri-oxidized phase MoO_3_ is identified by the doublet with components at 234 and 236.4 eV attributed to Mo3d_5/2_ and Mo 3d_3/2_, respectively (Figure 5a,b). The relative atomic concentration of oxides in the samples was ~1.0%. Notably, in the Mo3d region, no significant changes were observed in the XPS spectrum following 40 min of functionalization of the powder sample.

Additionally, the XPS analysis of the S2p core-level region reveals two intense peaks located at 162.8 and 164 eV, corresponding to 2p_3/2_ and 2p_1/2_ orbitals of the semi-conducting phase of 2H-MoS_2_ (Figure 5c,d). Figure 5f shows the XPS spectra recorded on the N1s region for the functionalized MoS_2_ powder; the components centered at ~398 and 402 eV are associated with N1s level overlapping with the Mo 3p_3/2_ level corresponding to Mo3p_3/2_ in MoS_2_ and in MoO_x_ at ~395.6 and 399 eV, respectively [41,42]. The additional components centered at 398 and 402 eV are associated with photoelectrons emitted from N1s levels in Mo–N and N–C [43,44,45]. The presence of nitrogen after the functionalization is associated with the use of nitrogen during the sputtering as a reactive gas.

**Figure 6:**
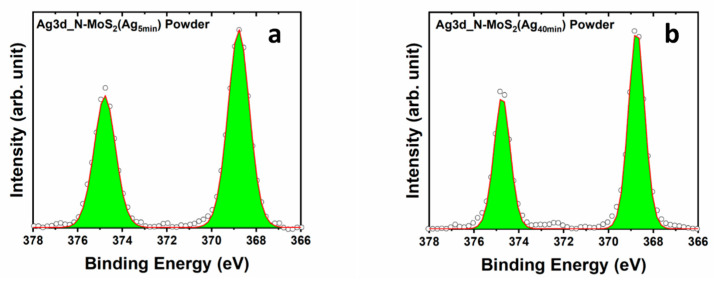
XPS core level of Ag3d regions of (**a**) N-MoS_2_ (Ag_5min_) powder and (**b**) N-MoS_2_ (Ag_40min_) powder showing the FWHM of Ag peaks of each functionalized sample.

In Figure 6, the Ag3d region is presented for powder samples functionalized with Ag nanoparticles for 5 min and 40 min. It is observed that as the deposition time increases, the Full Width at Half Maximum (FWHM) of the Ag3d peaks decreases from 1.14 to 0.86 eV. The presence of sharp peaks in the XPS spectrum indicates the bulk form of silver, while the broader peaks are characteristic of small silver nanoparticles [46]. The broadening of core-level XPS peaks for small nanoparticles can be attributed to the size effects and surface characteristics of the nanoparticles. In silver nanoparticles, the high surface-to-volume ratio results in a significant proportion of the atoms located at the surface or near-surface regions. These surface atoms are in modified electronic environments and interactions compared to those in the bulk material. The modified electronic structure of surface atoms leads to a distribution of binding energies for the emitted photoelectrons [47]. This distribution results in the broader peaks in the XPS spectrum of silver nanoparticles. In contrast, bulk silver or large particles have a more uniform and extended atomic arrangement, with a relatively constant electronic environment throughout the material, resulting in sharper XPS peaks [46]. Therefore, with increasing deposition time, the size of the particles increases, which agrees with the observation made by scanning electron microscopy (SEM), suggesting the growth of a nanoparticulated thin film (as evidenced in Supporting Figures S2 and S4). The relative atomic concentrations of silver and nitrogen were determined based on the XPS Data (Table 2).

**Figure 7:**
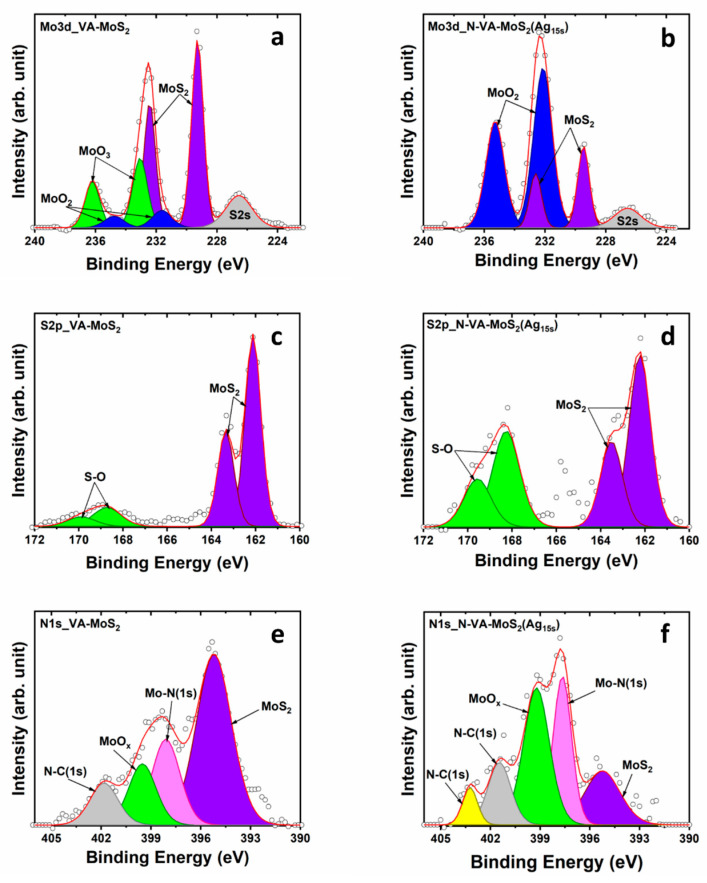
XPS core level of Mo3d, S2p, and N1s regions of VA-MoS_2_, the as-synthesized and the functionalized N-MoS_2_ (Ag_15s_). (**a**,**b**) Mo3d region, (**c**,d) S2p region, and (**e**,**f**) N1s region.

Figure 7 shows the core levels Mo3d, S2p, N1s, and Ag3d for VA-MoS_2_. The Mo3d region of the as-synthesized VA-MoS_2_ presented a relatively high concentration of the oxidized components of Mo. The components used to reproduce the Mo3d peak consist of a doublet with components centered at ~229.3 and 232.5 eV, attributed to 3d_5/2_ and 3d_3/2_ orbitals of the semi-conducting phase 2H-MoS_2_, respectively. The two doublets, corresponding to MoO_2_ and trioxide MoO_3_, have components at 231.7 and 234.8 eV and 233.1 and 236.2 eV, respectively. The Mo3d region becomes relatively dominated by the MoO_2_ doublet after 15 s of functionalization (Figure 7b). The relative atomic concentration of both oxides MoO_x_ (where x represents 2 or 3) in the as-synthesized sample first increased for increasing functionalization time from about 3.5% to 6.5% after 5 s, before decreasing to 5.0% for 10 s and 2.5% for 15 s of functionalization. This can be explained by a chemical etching occurring during the functionalization, as reported in the study by C. Lambaré et al. [48]. Analyzing the S2p region, the doublet with components centered at 162.1 and 163.3 eV, referred to as S2p_3/2_ and S2p_1/2_, respectively, is associated with 2H-MoS_2_. Additionally, the doublet at 168.7 and 169.9 eV is representative of S–O bonds. The relative intensity of this doublet increases compared to the 2H phase doublet (Figure 7c,d). The N1s region was reproduced using four singlets, at 395.2, 398.1, 399.5, and 402.0 eV, respectively, associated with the Mo3p_3/2_ peak of MoS_2_, the N1s peak of Mo–N bonds, the Mo3p_3/2_ peak of the MoO_x_ compound, and the N1s peak of carbon nitride [41,42,43,44,45]. The relative intensity of the component relative to MoS_2_ decreases after 15 s of plasma deposition, reflecting the incorporation of different species within the skeleton of the VA-MoS_2_. An additional peak at 403.2 eV arose after the functionalization process, attributed to carbon nitride (Figure 7e,f).

The grafting of oxygen during the functionalization may be influenced by the base pressure, which should not surpass 10^−6^ Torr to achieve optimal outcomes [49]. Nevertheless, despite maintaining a base pressure of 10^−6^ Torr, the generation of topographic defects during functionalization, which exhibit high reactivity to oxygen, and the lack of connection between the analysis chamber and the XPS analysis chamber suggest that the excess of oxygen detected could be attributed to exposure to atmospheric conditions during sample transfer between analysis and functionalization chamber.

**Figure 8:**
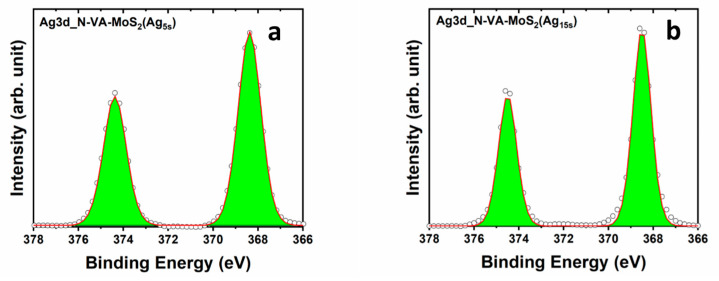
XPS core level of Ag3d regions of (**a**) N-VA-MoS_2_ (Ag_5s_) and (**b**) N-VA-MoS_2_ (Ag_15s_).

Concerning the Ag3d region, the same trend of decreasing FWHM of the emerged Ag peaks was revealed when comparing Ag peaks for functionalization time of 5 s and 15 s, which also occurred for the powder samples with increasing deposition time. The FWHM of the Ag3d doublet decreases from 1.2 eV for 5 s of deposition time to 0.9 eV for 15 s (Figure 8). More information is in Supporting Figures S3 and S4.

Table 3 resumes the relative atomic concentration of Ag and N for different functionalization times.

### 3.3. Valence Band Offset (VBO)

**Figure 9:**
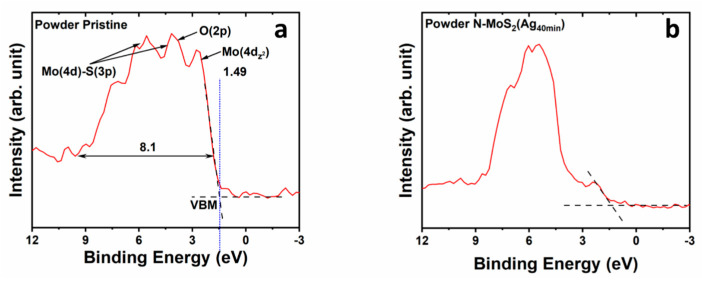
Valence bands of MoS_2_ powder samples, (**a**) the pristine powder with different orbitals hybridization showing VBM and VB ranging. (**b**) Powder N-MoS2(Ag40min) VBM is located at 1.44 eV, with Ag photoelectrons showing notably quasi-Ag VB.

The impact of silver nanoparticles (AgNPs) on the valence band of the samples is explored [50,51]. In Figure 9, the intercept of the baseline and the slope of the valence band edge reveals the valence band maximum (VBM) of the pristine powder located at 1.49 eV. The contributions of different orbitals, including Mo(4d_Z_^2^), Mo(4d)–S(3p) coupling, and O(2p), are identified. The observed valence band extends over ~8.0 eV. The Mo3d_5/2_ core level is centered at 229.9 eV, indicating a separation of 228.4 eV from the valence band maximum (VBM) in the pristine MoS_2_ powder. The functionalized sample N-MoS_2_(Ag_40min_) exhibited a slight variation of 0.2 eV in the Mo3d_5/2_ core level at 229.7 eV. Comparatively, the valence band maximum experienced a minor shift to 1.44 eV (Figure 9b).

**Figure 10:**
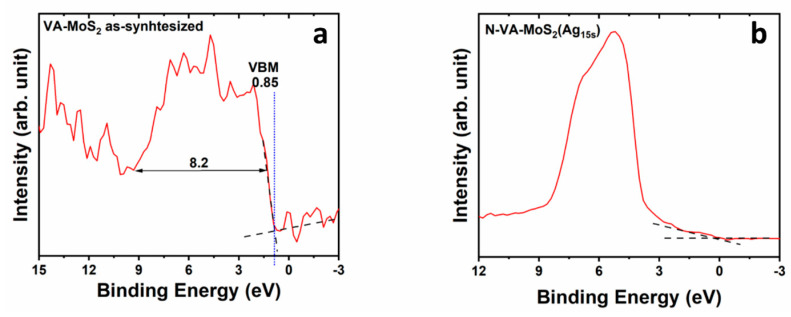
Valence band XPS spectra (red line) of (**a**) as-synthesized VA-MoS_2_ and (**b**) N-VA-MoS_2_ (Ag_15s_). Black lines indicate the top of the valence band.

Additionally, the signal attributed to Mo(4d_Z_^2^) photoelectrons became relatively low compared to photoelectrons from the Ag 4d shell. The Ag d-shell cross-section for photoemission is ten times larger than the Mo 4d shell for the used excitation X-ray photons [52]. Therefore, photoelectrons generated at the associated electronic level will make a bigger contribution to the valence band spectrum than the one generated at the Mo 4d-shell-associated levels. (Detailed XPS VB spectra for various functionalized powder samples are shown in Supporting Figures S5 and S6). Similarly, analogous behavior was observed when comparing VA-MoS_2_ before and after it was nitrogen-doped and silver-decorated (Figure 10). The VBM at 0.85 for the as-synthesized sample shifts to 0.3 eV for the sample functionalized for 15 s; the zero energy here represents the position of the Fermi level. This shift indicates a p-type MoS_2_ [53,54]. Surface modification through metal particle decoration can lead to a redistribution of surface states and the generation of a surface dipole moment [55,56,57,58].

## 4. Conclusions

This study focused on the comprehensive XPS investigation of silver-decorated vertically aligned MoS_2_-MoO_x_ nanosheets, emphasizing the morphology of MoS_2_, particularly vertically aligned MoS_2_. Vertically aligned MoS_2_ substrates were prepared by a CVD two-step sulfurization process, and silver decoration was performed using pulsed magnetron sputtering in the presence of nitrogen as a reactive gas. Characterization techniques such as X-ray photoelectron spectroscopy (XPS) and scanning electron microscopy (SEM) were utilized to analyze the samples. The presence of Ag(NPs) and nitrogen within the substrate structure was confirmed through XPS core-level analysis. Both commercial powder and VA-MoS_2_ reveal the semi-conducting MoS_2_ phase with a low amount of oxides. SEM images demonstrated the successful decoration of MoS_2_ with silver nanoparticles, demonstrating the impact of silver deposition time on the size and distribution of Ag(NPs) on the MoS_2_ surface. XPS core-level characterization exhibits a major change in the chemical structure of the VA-MoS_2_ after the deposition of silver particles. In comparison, the commercial powder demonstrates more stability in terms of chemical changes after functionalization. The slight valence band offset alteration indicated changes in the VBM based on functionalization time for both the commercial powder and VA-MoS_2_.

## Figures and Tables

**Table 1:** Ag(NPs) size average of the functionalized MoS_2_ powder and VA-MoS_2_ in nm.

Powder	Ag(NPs) Size Average (nm)	VA-MoS_2_	Ag(NPs) Size Average (nm)
N-MoS_2_ (Ag_5min_)	8.2 ± 1.0	N-VA-MoS_2_ (Ag_5s_)	6.7 ± 0.2
N-MoS_2_ (Ag_10min_)	13.4 ± 0.8	N-VA-MoS_2_ (Ag_10s_)	26.4 ± 2.3
N-MoS_2_ (Ag_15min_)	16.6 ± 1.0	N-VA-MoS_2_ (Ag_15s_)	*
N-MoS_2_ (Ag_20min_)	18.0 ± 0.9		
N-MoS_2_ (Ag_40min_)	36.4 ± 2.7		

* The size of the NPs on the sample functionalized for 15 seconds was unattainable since they formed a particulate thin film, rendering the measurement unfeasible.

**Table 2:** Atomic concentration of Ag and N for different samples for different functionalization times, as obtained by XPS Data.

Materials	Ag (%)	N (%)
N-MoS_2_ (Ag_5min_)	1.3 ± 0.06	2.1
N-MoS_2_ (Ag_10min_)	4.1 ± 0.04	2.2
N-MoS_2_ (Ag_15min_)	4.1 ± 0.04	2.3
N-MoS_2_ (Ag_20min_)	7.0 ± 0.07	3.2
N-MoS_2_ (Ag_40min_)	10.4 ± 0.10	4.0

**Table 3:** Atomic concentration percentages of Ag and N for N-VA-MoS_2_ different samples as the functionalization time increases, as obtained by XPS Data.

Materials	Ag (%)	N (%)
N-VA-MoS_2_ (Ag_5s_)	13.8	13.8
N-VA-MoS_2_ (Ag_10s_)	23.3	10.3
N-VA-MoS_2_ (Ag_15s_)	31.3	7.7

## Data Availability

The original contributions presented in the study are included in the article/Supplementary Materials, further inquiries can be directed to the corresponding author.

## Supplementary Materials

The following supporting information can be downloaded at: https://www.mdpi.com/article/10.3390/ma17122882/s1, Figure S1: SEM image recorded in a cross-section of sample VA-MoS2 decorated with Ag(NPs) for 10s (a), (b) magnification of figure (a). Figure S2: XPS Ag3d regions for different functionalized powder samples show the evolution of Ag peaks for (a) 5 min of deposition, (b) 10 min of deposition, (c) 15 min of deposition, (d) 20 min of deposition, and (e) 40 min of deposition; Figure S3: XPS Ag3d regions for different functionalized VA samples show the evolution of Ag peaks for (a) 5 s of deposition, (b) 10 s of deposition, and (c) 15 s of deposition; Figure S4: variation of FWHM of Ag peaks in Ag3d region according to the data collected by XPS. (a) shows the decrease in the FWHM of the peaks of powder samples after different plasma deposition times, from 1.14 for t = 5 min to 0.86 eV for t = 40 min. (b) reveals a decrease in the FWHM of the VA samples from 1.2 for t = 5 s to 0.96 for t = 15 s. Figure S5: XPS Valence band for powder samples. (a) The pristine powder, (b), (c), (d), (e), and (f) for the functionalized samples for 5, 10, 15, 20, and 40 min, respectively. Figure S6: XPS Valence bands of different VA samples. (a) The as-synthetized VA-MoS_2_, (b), (c), and (d) for the functionalized samples for 5, 10, and 15 s, respectively.
