# Supplementary material for: Silver Decoration of Vertically Aligned MoS2-MoOx Nanosheets: A Comprehensive XPS Investigation
Source: Materials (Basel). 2024 Jun 13;17(12):2882. doi: 10.3390/ma17122882 (PMC11205143; doi:10.3390/ma17122882)
Supplement: Supplementary file 1 [file materials-17-02882-s001.zip › materials-3042194-supplementary.pdf]

Ag/N one pot functionalization effects on vertically aligned MoS_2

(XPS detailed study)

Supporting Information

Khaled Al Youssef, Arkaprava Das*, Jean-François Colomer[ȣ], Axel Hemberg[T], Xavier Noirfalise[T], Carla Bittencourt**

*Chimie des interactions Plasma-Surface (ChIPS), Materials Institute, University of Mons
23 Place du Parc, 7000 Mons, Belgium*

[T]*Materia Nova, 3 Avenue Copernic, 7000 Mons, Belgium*

[ȣ]*Research Group on Carbon Nanostructures (CARBONNAGe), University of Namur
61 Rue de Bruxelles, 5000 Namur, Belgium*

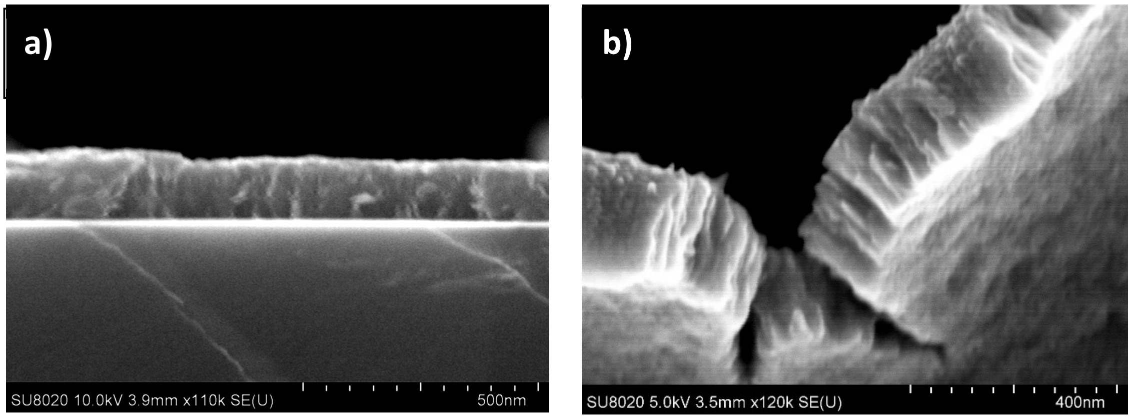

Figure S1: SEM image recorded in a cross-section of sample VA-MoS2 decorated with Ag(NPs) for 10s (a), (b) magnification of figure (a)

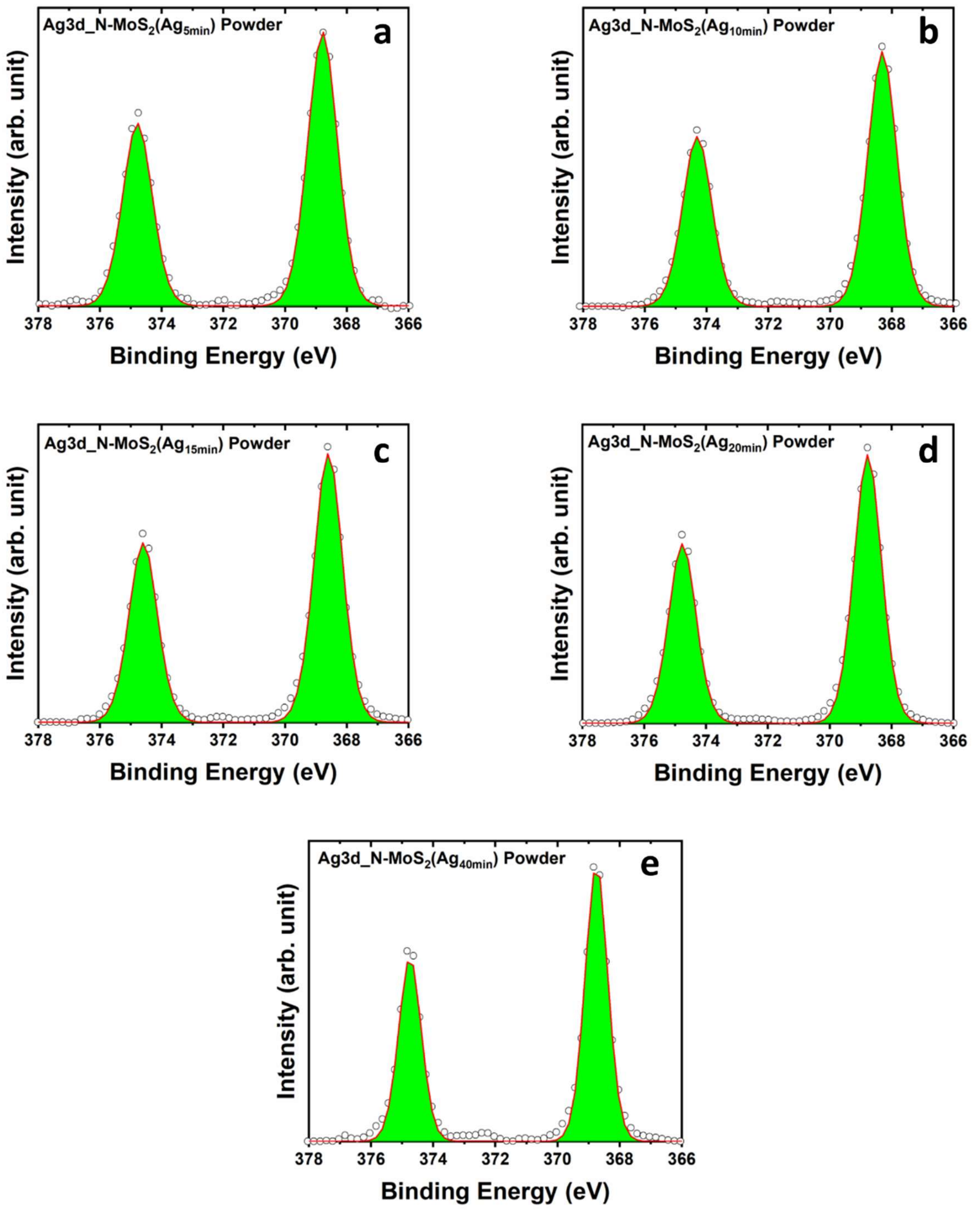

Figure S2: Ag3d regions for different functionalized powder samples show the evolution of Ag peaks for (a) 5 min of deposition, (b) 10 min of deposition, (c) 15 min of deposition, (d) 20 min of deposition, and (e) 40 min of deposition.

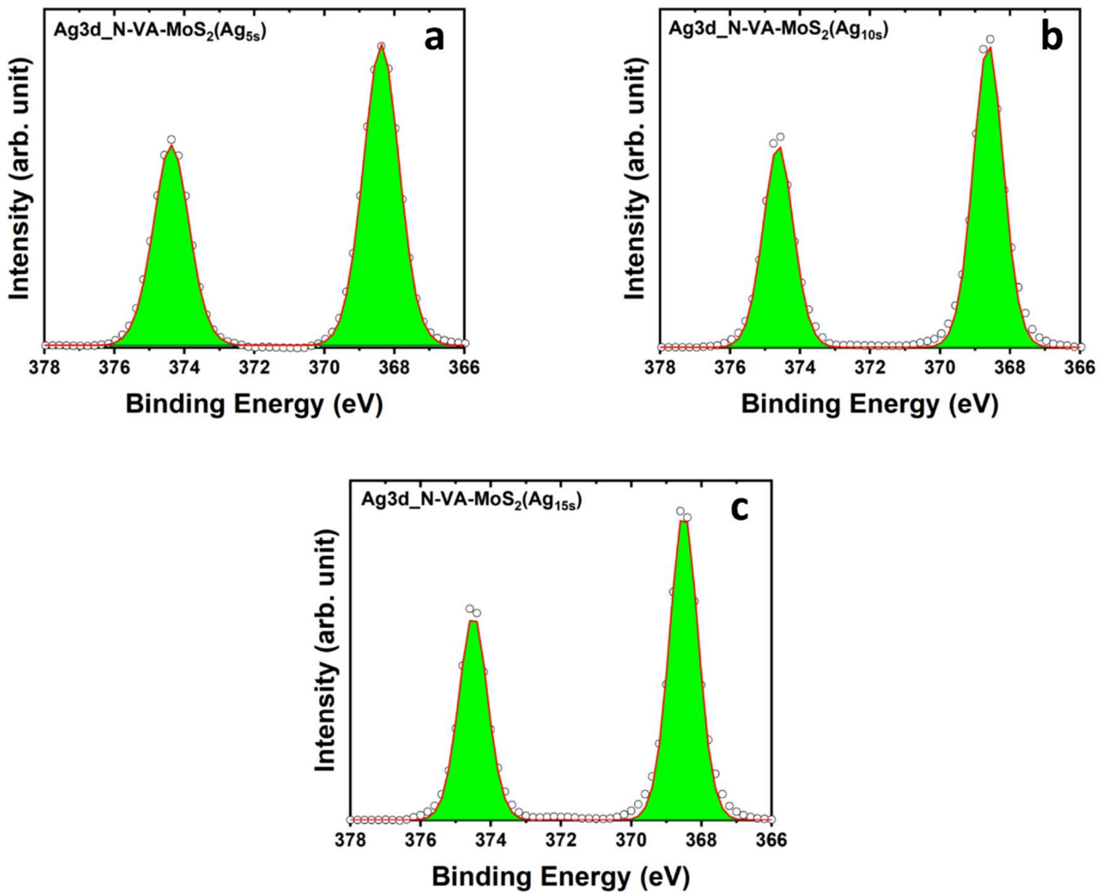

Figure S3: Ag3d regions for different functionalized VA samples show the evolution of Ag peaks for (a) 5 s of deposition, (b) 10 s of deposition, and (c) 15 s of deposition.

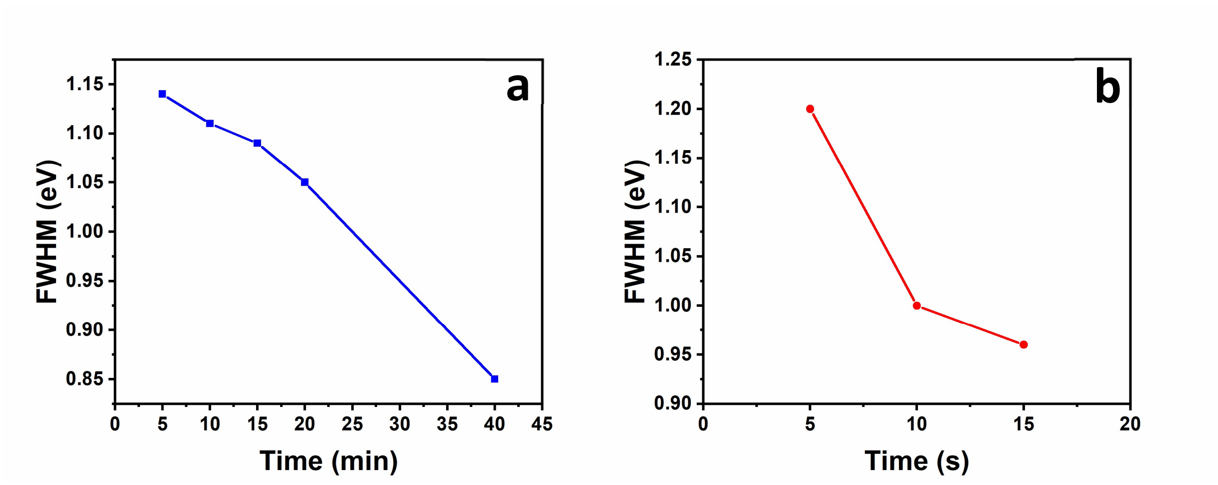

Figure S4: Variation of FWHM of Ag peaks in Ag3d region according to the data collected by XPS. (a) shows the decrease in the FWHM of the peaks of powder samples after different plasma deposition times, from 1.14 for t = 5 min to 0.86 eV for t = 40 min. (b) reveals also a decrease of the FWHM of the VA samples from 1.2 for t = 5 s to 0.96 for t = 15 s.

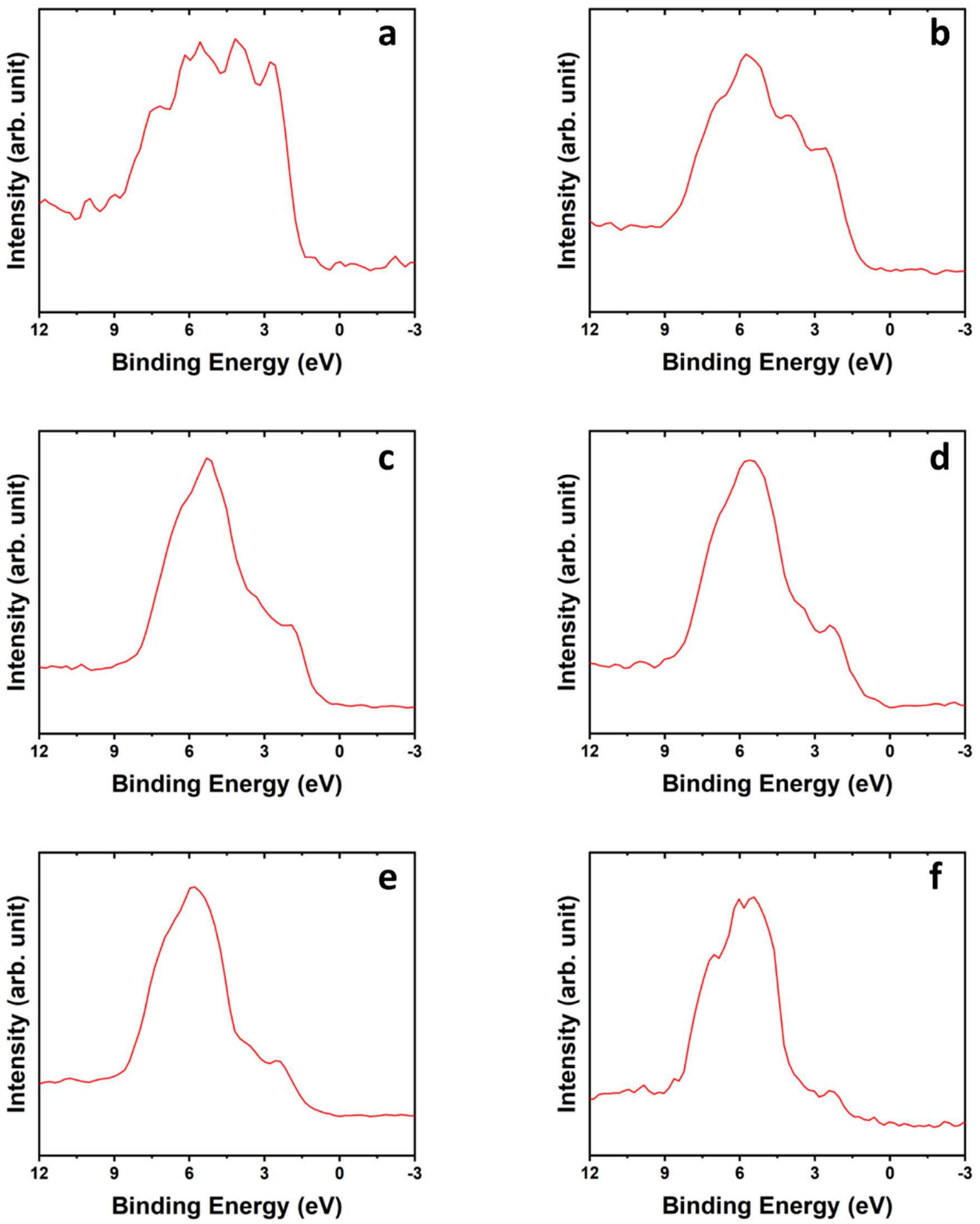

Figure S5: Valence bands of different powder samples. (a) the pristine powder, (b) (c) (d) (e) and (f) for the functionalized samples for 5, 10, 15, 20 and 40 min respectively.

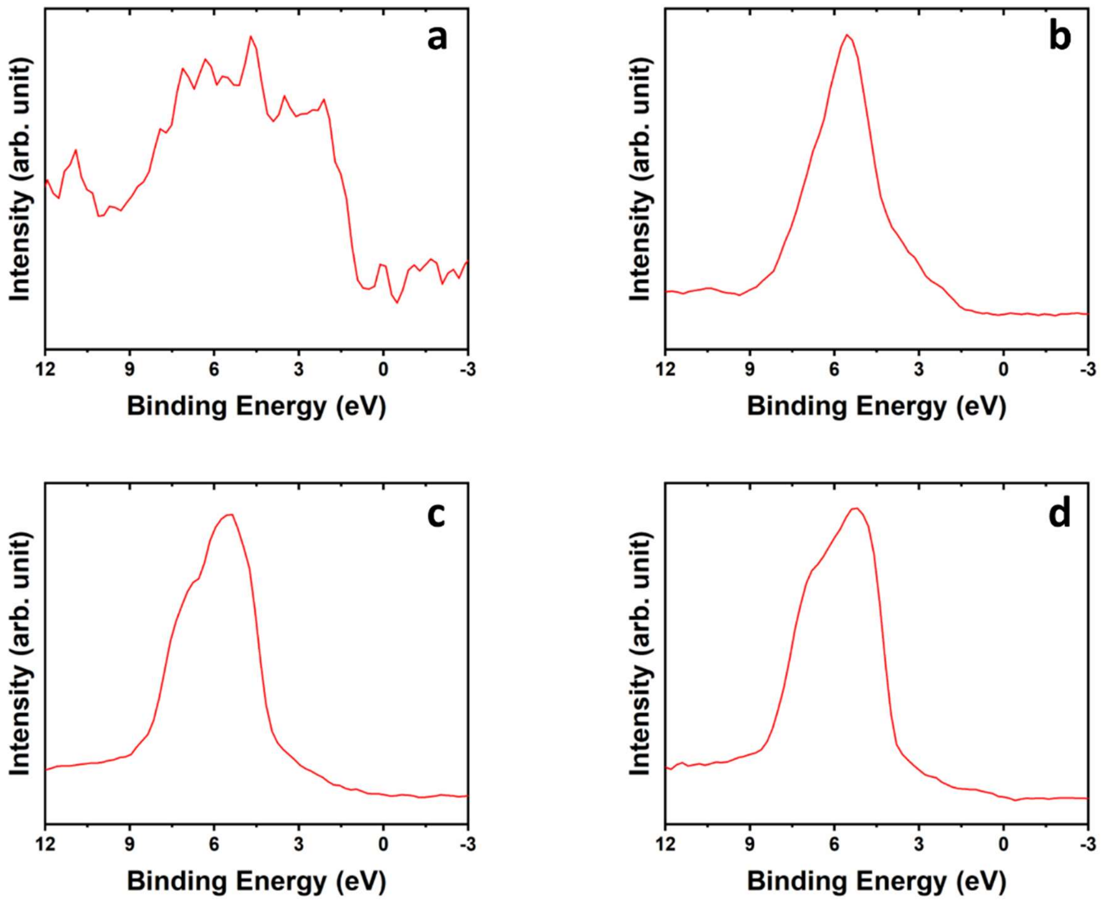

Figure S6: Valence bands of different VA samples. (a) the as-synthetized VA-MoS_2, (b) (c), and (d) for the functionalized samples for 5, 10, and 15 s, respectively.
